# Successful endovascular therapy for an adolescent patient with cerebral venous sinus thrombosis: A case report

**DOI:** 10.1097/MD.0000000000027024

**Published:** 2021-09-03

**Authors:** Tao Peng, Zini Zhang, Bifeng Zu, Bitang Dan

**Affiliations:** a Department of Neurology, the Third People's Hospital of Hubei Province, Wuhan, PR China; b Department of Neurology, Zhongnan Hospital, Wuhan University, No.169. Donghu Road, Wuchang District, Wuhan, PR China.

**Keywords:** anti-coagulant therapy, cerebral venous sinus thrombosis, endovascular therapy, protein S deficiency

## Abstract

**Rationale:**

Cerebral venous sinus thrombosis associated with protein S deficiency is rare in adolescent patients and has high disability and fatality.

Patient concerns: A 15-year-old male student presented in the hospital with sudden headache, nausea, and vomiting and was diagnosed with protein S deficiency by gene testing.

**Diagnoses:**

Cerebral venous sinus thrombosis due to protein S deficiency was diagnosed in this adolescent patient, who underwent successful endovascular therapy (EVT).

**Interventions:**

The patient was treated with standard anti-coagulation therapy including low-molecular-weight heparin (90 IU/kg/Q12 h) and dehydrant (mannitol 125 mL Q8 h); however, the symptoms were not alleviated. Successful EVT was implemented.

**Outcomes:**

Both the superior sagittal sinus and bilateral transverse sinus were recanalized after thrombus clearance. The patient achieved a complete recovery without any other stroke recurrence during follow-up.

**Lessons:**

EVT can be performed with favorable and effective clinical outcomes in adolescent cerebral venous sinus thrombosis patients with protein S deficiency. EVT associated with standard anti-coagulation therapy may improve the prognosis and reduce mortality among such patients.

## 1 Introduction

Cerebral venous sinus thrombosis (CVST) is a rare cerebrovascular disease. The annual incidence in adults is 1.32/100,000, and that in females 31 to 50 years of age is considerably higher (2.78/100,000).^[1,2]^ However, the incidence among children under 18 years of age is 0.67/100,000, and most of those affected are neonates.^[3]^ The clinical characteristics of CVST are diverse, leading difficulties and delays in diagnoses. Although guidelines generally recommend anti-coagulation therapy as the basic treatment for CVST, the outcomes of nearly 20% of adult patients are disability or death, and nearly 47% of adolescent patients have a poor prognosis after treatment.^[4]^ With the development of endovascular therapy (EVT) for cerebral arterial diseases, EVT has become a salvage treatment for such patients. Although several single-center studies have reported good outcomes, a multicenter, prospective, randomized, controlled study is still necessary. Moreover, reports of adolescent patients with CVST who have undergone successful EVT are even rarer. Here, the case of a comatose adolescent patient with CVST who was successfully treated by EVT is described.

## 2 Case report

**Figure 1:**
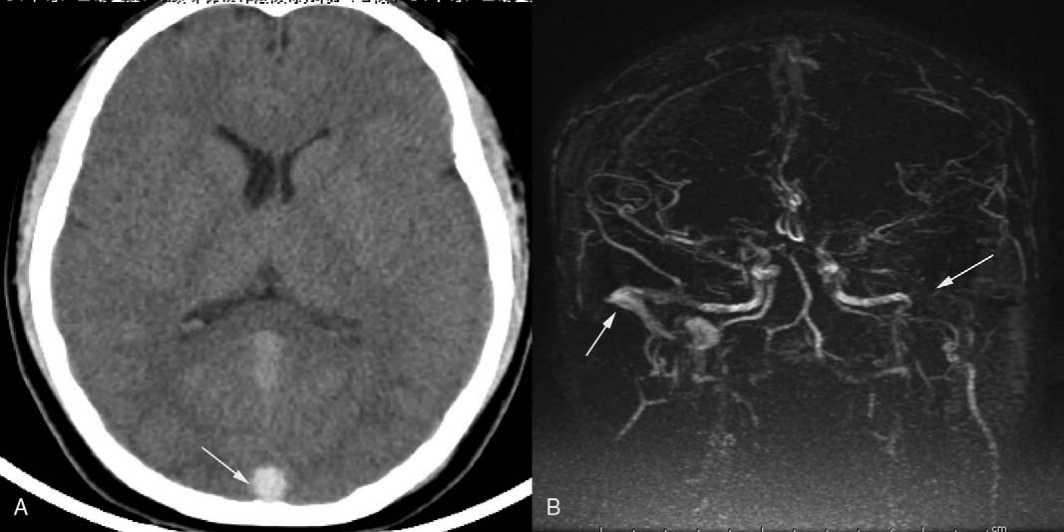
A thrombus sign in the SSS (white arrow) observed on CT; MRV showing a normal flow signal in the right transverse sinus and sigmoid sinus, but non-visualization of the left transverse and sigmoid sinus (white arrows). CT = computerized tomography, MRV = magnetic resonance venography, SSS = superior sagittal sinus.

**Figure 2:**
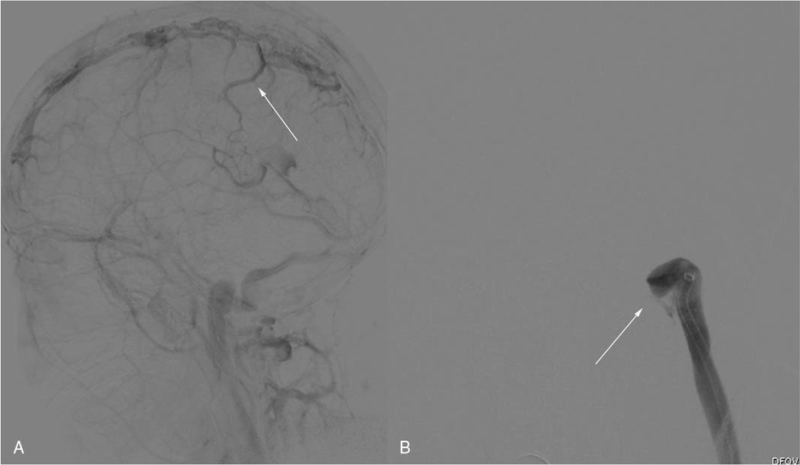
A. DSA showing poor visualization of the SSS and cortical venous reflux (white arrow); B. Occlusion of the left transverse sinus (white arrow). DSA = digital subtraction angiography, SSS = superior sagittal sinus.

**Figure 3:**
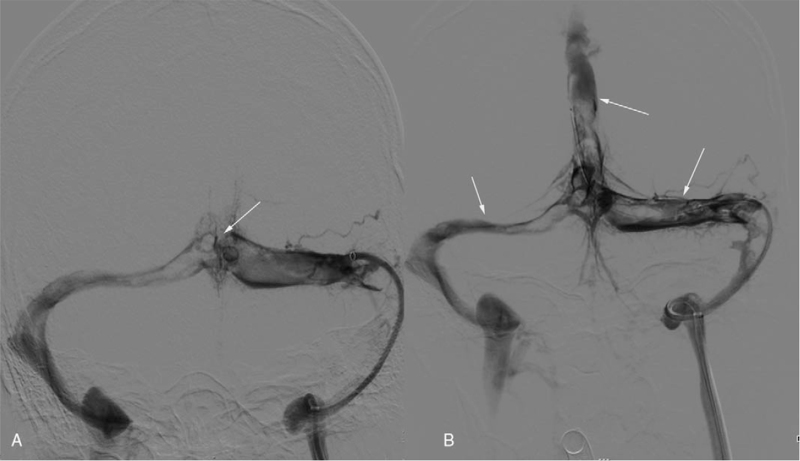
A. A normal flow signal in bilateral transverse sinus. Occlusion of the SSS (white arrow); B. A normal flow signal in the SSS, bilateral transverse sinus, and sigmoid sinus. SSS = superior sagittal sinus.

**Figure 4:**
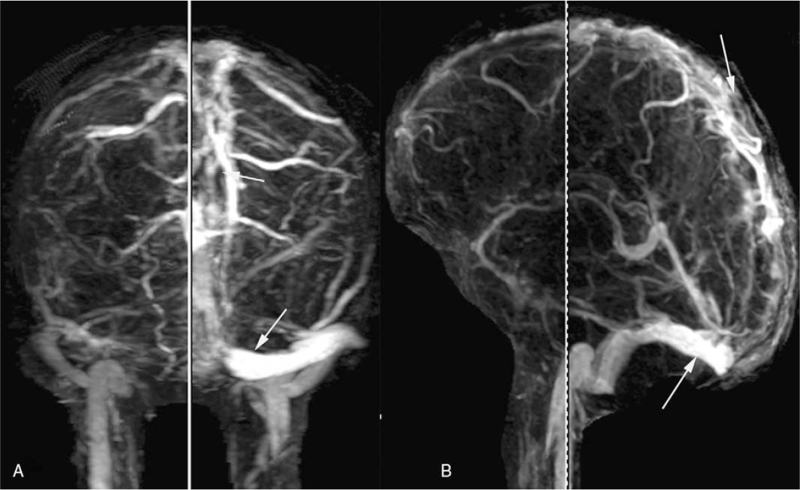
MRV obtained 1 week after the procedure, showing clear visualization of the SSS and right transverse sinus. MRV = magnetic resonance venography, SSS = superior sagittal sinus.

A 15-year-old male student presented in the local hospital with a 2-day history of sudden severe headache, nausea, and vomiting. He was previously healthy without a previous medical history of cerebral arterial diseases. Cranial computerized tomography showed hyperattenuation in the superior sagittal sinus (SSS) and no intracranial hemorrhage (ICH) (Fig. 1-A). Magnetic resonance venography confirmed the diagnosis, with non-visualization of the left transverse sinus and poor visualization of the right transverse sinus (Fig. 1-B). After anti-coagulant treatment with low-molecular-weight heparin (90 IU/kg/Q12 h) and dehydrant (mannitol 125 mL Q8 h), the symptoms were not alleviated. After admission to our hospital, he was in a shallow coma with an intracranial pressure of 420 mmH_2_O. EVT was performed immediately due to the poor response to anti-coagulant treatment and deteriorating symptoms. Digital subtraction angiography showed SSS occlusion and cortical venous distension (Fig. 2-A). Left transverse sinus occlusion was observed on descending phlebography (Fig. 2-B). A long sheath was used to suction the thrombus in the bilateral transverse sinus (Fig. 3-A). A stent was used to help suction the thrombus in the distal SSS, and a percutaneous angioplasty balloon was used to dilate the proximal SSS. Both the SSS and bilateral transverse sinus were recanalized after the clearance of the thrombus (Fig. 3-B). Postoperative magnetic resonance venography showed normal SSS and no occlusion in the right transverse sinus (Fig. 4-A and B).

The reason for CVST in this patient was protein S deficiency, which led to a reduction in protein activity to 20.5% (normal range 63.5%–149%). Genetic testing revealed a PROS1 mutation. After EVT and anti-coagulation therapy (ACT) treatment, the patient recovered uneventfully without any symptoms. The patient was discharged 1 week after EVT without any neurological deficits.

Access to patient records for data collection and analysis of the data were approved by our local medical ethics committee. The patient and his father provided informed consent for their clinical data to be used anonymously for research purposes.

## 3 Discussion

Although ACT generally remains the first treatment for CVST recommended by many guidelines, 20% of patients still end up with disability or death. As demonstrated by some studies, the risk factors for these patients include mental disorder, a comatose state, ICH, and deep cerebral venous thrombosis.^[5]^ According to Sebire et al,^[6]^ coma is a significant predictor of death among CVST patients. Siddiqui et al^[7]^ reported that 185 adults with severe CVST underwent EVT, 84% of patients had a good prognosis, and 12% died, suggesting that EVT might improve the prognosis for adults with severe CVST. Liao et al^[8]^ obtained similar outcomes in patients with CVST who underwent EVT. They found that a clot burden (>10 cm length) involving SSS was associated with a poor prognosis and that EVT might be a salvage treatment in severe CVST patients with a poor response to ACT. Although many single-center studies and individual case reports have proposed good outcomes in patients with CVST who underwent EVT, there were no sufficient prospective, randomized, controlled studies to support the efficacy and safety of EVT in the subgroup analysis. One prospective, multicenter, randomized, controlled trial showed that EVT and standard medical treatment did not improve the outcomes in severe CVST patients compared with standard medical treatment.^[9]^ There were several possible reasons for the poor outcomes in the study: the EVT technique in that period was not standard due to the lack of devices; the number of patients enrolled in this study was small, and insufficient data were collected. A meta-analysis conducted by Ilyas et al^[10]^ indicated that EVT was an effective approach to treat critical CVST patients. However, endovascular mechanical thrombectomy combined with medical treatment did not result in additional harm or benefit. Further studies are necessary to prove the benefits of EVT in severe CVST patients. Based on the literature, although several single-center retrospective studies have noted good prognoses in severe CVST patients who underwent EVT, multicenter prospective studies and meta-analysis results did not support the superiority of EVT over standard treatment. Possible reasons might be that the majority of CVST patients were not in critical condition and that standard anti-coagulation had relatively good outcomes. In addition, the insufficient number of critical CVST patients resulted in a questionable result. More studies should be carried out in the future to verify the efficacy and safety of EVT in patients with CVST.

ACT has been recommended by European guidelines as the preferred treatment for CVST. However, these guidelines are based on treatment outcomes among adults, and clinical studies have not yet proven the efficacy of ACT and EVT in adolescent patients. ACT can effectively prevent the continuation of venous thrombosis, promote collateral blood circulation, and improve the hypercoagulable state with a very simple operation. However, anti-coagulant therapy cannot dissolve the old thrombus, and the effect is slower, making it not suitable for patients with severe CVST. ACT might improve the prognosis among adolescents with CVST, but the improvement is not significant.^[6]^ Moharir et al^[4]^ reported that 85 CVST adolescent patients received ACT. Among them, 6 had major hemorrhage (5 with known ICH before treatment). ACT-associated hemorrhage is non-fatal, and 50% of patients had a poor prognosis, with the control group having a similar percentage. Early radiographic follow-ups showed thrombi in 17 patients (17/54) without ACT and in 4 (4/66) with ACT. A thrombus as a venous entity might lead to an unfavorable prognosis. Non-treatment was associated with thrombus propagation, as observed in 1/4 of neonates with CVST and 1/3 of children with CVST in the control group. Therefore, more attention should be given to ACT in CVST, and the efficacy of EVT in adolescent CVST patients remains unclear. Koji et al^[11]^ reviewed the data of 25 adolescent patients in 6 studies from 2008 to 2016, and the mean onset age was 14 years. Cerebral hemorrhage was observed in 2 cases (8%). In terms of Modified Rankin Scale scores generated 3 months post-EVT, 16 patients (64%) scored 0 to 2, 4 (16%) scored 3 to 5, and 5 (20%) died. Compared with those of the adult patients, the prognosis and mortality rates of the adolescent patients were less favorable, while the rates of ICH and catheter-associated complications were equivalent. Unfavorable prognoses in adolescent CVST patients might be associated with capillary underdevelopment, which may cause the failure of recanalization by increased cerebral blood flow after anastomosis between meningeal and other vessels.^[12]^ In a study involving 3 adolescents with severe CVST, children and young adults had lower tolerance to CVST than adults. The clinical course was highly variable without long-term stability. EVT, which is a new salvage treatment for CVST, can quickly open occluded vessels, promote venous blood return, effectively reduce intracranial hypertension, and quickly relieve the state of illness. EVT should be performed in a comatose state to improve outcomes. Therefore, comatose adolescents with CVST should undergo EVT as soon as possible.^[11]^ However, EVT has high technical requirements without a special device for venous sinus thrombectomy, so this procedure should be performed in an experienced center.

Protein S deficiency is a hereditary thrombophilia with a familial prevalence between 0.03% and 0.13%.^[13]^ The condition has been divided into autosomal dominant inheritance and recessive inheritance. The former often leads to onset in adults, while the latter in infants and young children affects the eyes, blood vessels, lungs, and skin, with a severe onset. In this patient with protein S deficiency, genetic testing revealed a PROS1 mutation at location chr3:93692761 and 1 heterozygous mutation at c-168>T. The patient was heterozygous, which fit the dominant inheritance pattern of the condition recorded in the Human Gene Mutation Database. Protein S deficiency increased the possibility of venous thrombosis. The heterozygous mutation triggered the onset of venous thrombosis in this patient. After the diagnosis of CVST and treatment with adequate ACT (low-molecular-weight heparin) and dehydrant (mannitol), the patient deteriorated quickly and developed a consciousness disorder. Severe CVST was confirmed, and EVT was performed by recanalization of the SSS and left transverse sinus. After the procedure, the patient soon regained consciousness, and his headache was greatly alleviated. Standard ACT with warfarin was used in this patient. The patient recovered well with no thrombosis recurrence.

According to the guidelines, warfarin is recommended for CVST caused by thrombophilia, including protein S deficiency. To date, some studies have identified new anti-coagulants, and warfarin showed no significant difference in terms of efficacy or risk of bleeding events in treating venous thrombosis.^[14]^ Some studies have even reported that using new anti-coagulants in patients with inherited thrombophilia achieved outcomes similar to those of warfarin with lower bleeding risk.^[15]^ Some clinical reports have found that a standard dosage of new anti-coagulants showed less favorable results in treating CVST due to protein S deficiency, but satisfactory results were attained by increasing the dosage.^[16]^ The use of new anti-coagulants in treating inherited thrombophilia remains controversial, and more studies are necessary.

## 4 Conclusions

EVT can be performed with favorable and effective clinical outcomes in adolescent CVST patients. EVT associated with standard ACT may improve the prognosis and reduce mortality in such patients.

## Author contributions

**Investigation:** Tao Peng.

**Methodology:** Bifeng Zu.

**Project administration:** Bitang Dan.

**Software:** Tao Peng, Bifeng Zu.

**Validation:** Bitang Dan.

**Writing – original draft:** Tao Peng, Bitang Dan.

**Writing – review & editing:** Zini Zhang, Bitang Dan.
